# Ultrasound-detected lateral band snapping syndrome in proximal interphalangeal joint of small finger—A rare case report

**DOI:** 10.1016/j.ijscr.2019.08.018

**Published:** 2019-08-20

**Authors:** Tyng-Shiuan Hsieh, Yi-Jie Kuo, Yu-Pin Chen

**Affiliations:** a School of Medicine, College of Medicine, Taipei Medical University, Taipei, Taiwan; b Department of Orthopaedic Surgery, Wan Fang Hospital, School of Medicine, College of Medicine, Taipei Medical University, Taipei, Taiwan

**Keywords:** Lateral band snapping syndrome, Ultrasound, Case report

## Abstract

**Background:**

Snapping fingers resulting from flexor tendon tenosynovitis at the metacarpophalangeal (MCP) joint, is common. However lateral band snapping syndrome (LPSS) in the proximal interphalangeal joint (PIPJ) are extremely rare.

**Case presentation:**

A 43-year-old female was diagnosed with LPSS in the PIPJ of fifth finger which was confirmed by dynamic evaluation upon ultrasound. Repair of retinacular ligament of the extensor tendon was performed. At the six-month follow up, the patient had regained full ROM with no discomfort, without evidence of recurrence.

**Discussion:**

The diagnosis of LPSS at the PIPJ is challenging. Dynamic evaluation of the extensor tendon over PIPJ is necessary to diagnose LBSS. Ultrasound was used to assess the movement of the lateral band of the extensor tendon when the fingers flexed and to demonstrate dynamically the snapping and subluxation of the lateral band, so as to facilitate confirming the diagnosis of LBSS.

**Conclusion:**

Given clinical suspicion of LBSS, we recommend ultrasonography as a feasible tool to confirm the diagnosis. Patients with LBSS may thus benefit from prompt repair of the retinacular ligament without sequela.

## Highlights

• The lateral band snapping syndrome (LBSS) in the proximal interphalangeal joint (PIPJ) is the subluxation of lateral band of the extensor tendon resulting from injury of the retinacular ligament, causing finger snapping during flexion of the PIPJ.
• Ultrasonography can visualize the subluxation of lateral band, facilitating diagnosis of LBSS in the PIPJ.
• Patients with LBSS may benefit from prompt repair of the retinacular ligament without sequela.

## 1 Introduction

Snapping fingers resulting from flexor tendon tenosynovitis at the metacarpophalangeal (MCP) joint is common. However other causes and other locations of snapping on the fingers are extremely rare. To date, few cases of snapping fingers at the proximal interphalangeal joint (PIPJ) have been reported. The method for diagnosing snapping fingers relies more on physical examination than on specific image studies [1]. This report presents a case of snapping syndrome at the PIPJ of the small finger. In the initial survey in this unique case, ultrasound was used to evaluate the dynamic movement of the extensor tendon and evaluate the instability of the lateral band. To the best of our knowledge, this is the first case report to include ultrasonographic imaging of lateral band snapping syndrome (LBSS) at the PIPJ. Our work has been reported in line with the SCARE criteria [2].

## 2 Case report

**Fig. 1:**
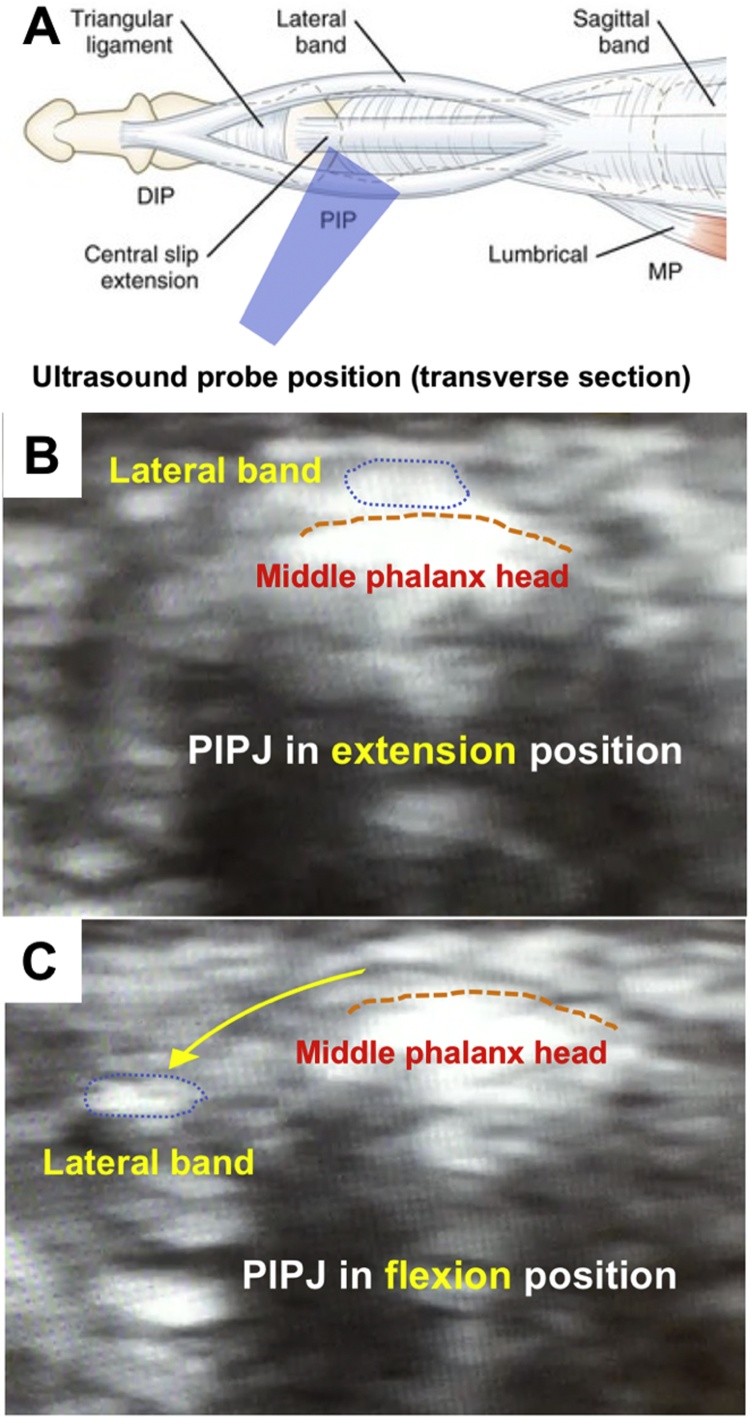
Dynamic change of lateral band under ultrasonography (A: Probe placement over dorsal and radial side of middle phalanx head as transverse section; B: Lateral band in situ over middle phalanx head upon extension of PIPJ; C: Subluxation of lateral band to radial side upon flexion of PIPJ.

A 43-year-old female with no specific underlying disease had been bothered by symptoms of finger snapping at the PIPJ of her right small finger for more than a decade. She denied any specific episode of trauma or previous surgical history with respect to the right small finger. Upon examination, no gross abnormality over the right small finger was observed and the finger exhibited a full range of motion over the PIPJ. However, crunching of the tendon could be found over the dorsal medial side of the PIPJ during flexion of the fifth PIPJ. Ultrasonography was then utilized to evaluate the dynamic change of the extensor mechanism with a broadband 7.5–10 MHz linear array scan head with the linear transducer placed transversely and longitudinally on dorsal medial side of the fifth PIPJ. (Acuson X600, Siemens, Germany) In dynamic imaging during flexion of the right fifth PIPJ from extension, subluxation of the lateral band of the extensor tendon to the radial side causing snapping symptoms were detected under the transverse section (Fig. 1) (Video 1).

**Fig. 2:**
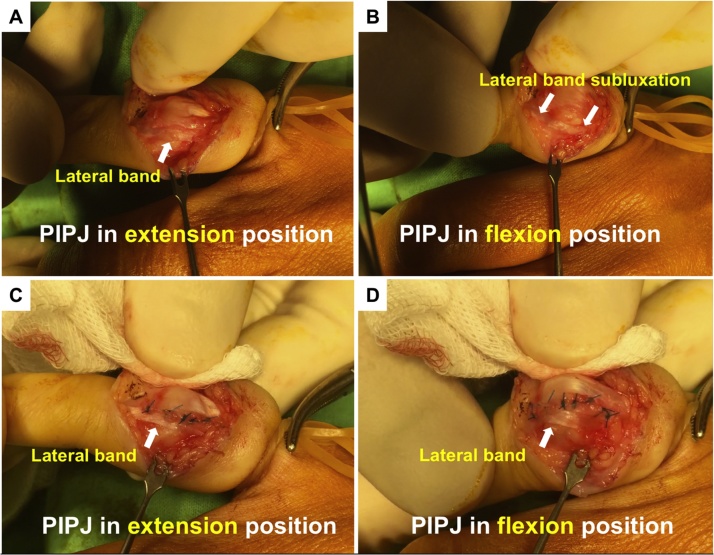
Surgical finding of subluxation of lateral band (A and B: Subluxation of lateral bands upon flexion of PIPJ before repair of ruptured retinacular ligament; C and D: Lateral band is located stably in situ after repair of ruptured retinacular ligament).

Exploration of the PIPJ with a curve incision under local anesthesia revealed rupture of the retinacular ligament with partial fibrosis union, laxity and lateral subluxation of the radial side of the lateral band with partial joint capsule rupture over the dorsal PIPJ (Fig. 2). The joint capsule was then repaired and the subluxation of the lateral band was sutured back to the retinacular ligament of the extensor tendon over the dorsal side of the PIPJ with 5-0 Prolene. Postoperatively, the small finger was immobilized in finger orthosis with full extension of the fingers for two weeks; then alternative switching of finger orthoses with full extension at night and restricted flexion in the day was applied for another four weeks. Unrestricted full active motion was allowed after the sixth week.

At the six-month follow up, the patient had regained full ROM with no discomfort, without evidence of recurrence.

## 3 Discussion

The PIPJ extensor mechanism involves three ligaments. The lateral band is controlled and protected by the transverse retinacular ligament and the triangular ligament. The transverse retinacular ligament prevents lateral band dorsal subluxation upon PIPJ extension. The triangular ligament prevents lateral band volar subluxation during PIPJ flexion. Since the triangular ligament and the transverse retinacular ligament are important for the function of the lateral band, the integrity and elasticity of these three ligaments are crucial to the functioning of PIP joint. As in the present case, an injury of the retinacular ligament can cause functional instability of the finger.

Yamaguchi et al. identified two common causes of snapping finger based on their operative findings. One is the formation of a tumor at the flexor pollicis longus at the MCP joint tendon sheath; the other is flexor tendon tendovaginitis in the thumb, middle finger or ring finger. These causes of the snapping finger are very common. However, a snapping finger at the PIP joint that arises from an injury of the extensor mechanism is extremely rare and only three cases have been reported in English [1,3,4].

One case involved snapping of the left small finger at the PIPJ as a result of post-traumatic retinacular ligament injury in a 24 year-old man [1]. Another case was due to solitary periosteal chondroma, impinging between the basal phalanx and extensor tendon in a 37 year-old man. The chondroma caused pain and a snapping ring finger at the PIP joint. [4] The third case involved a 14 year-old girl, whose snapping was due to both congenital ulnar declination at the PIPJ and a minor trauma that had caused the rupture of transverse retinacular ligament [3].

The diagnostic tools in the three cases were the plain film [1,3] and the CT scan [4]. The obvious abnormalities that were detected by the CT scan and the plain film were the chondroma and a congenital ulnar declination of 13 degree, respectively. However, subluxation of the lateral band and rupture of the retinacular ligament cannot be identified using the plain film. Therefore, in this case, ultrasound was used to assess the movement of the lateral band of the extensor tendon when the fingers flexed and to demonstrate dynamically the snapping and subluxation of the lateral band to the radial side, so as to confirm the diagnosis of LBSS.

Although snapping at the PIPJ is a specific symptom of LBSS, the other potential differential diagnosis may also include lateral band rupture of extensor tendon or ganglion cyst of tendon sheath. However, all of the tentative differential diagnosis can be excluded using ultrasonography [5]. Ultrasonography operates dynamically in real time; its low-cost, and provides high-resolution images. With the assistance of ultrasound, the lateral band rupture may illustrate abnormal lateral band with irregularity and hypoechogenicity. On the other hand, the ganglion cyst of tendon sheath may display an anechoic mass lesion. When used for proper indications, ultrasonography has a definite role in the diagnosis of injuries of the ligaments on small finger joints [6].

## 4 Conclusion

The case reported here demonstrates that when snapping or instability of the PIPJ of the fingers is present, LPSS, which may be benefit from surgical repair, should be listed as a differential diagnosis. Ultrasonography can be a reliable tool for confirming the anatomical and dynamic functional instability of finger extensor mechanisms.

## Sources of funding

No funding for our research.

## Ethical approval

The ethical approval was registered as TMU-JIRB N201810021.

## Consent

Informed consent for publication, including any necessary pho-tographs, has been obtained from the patient.

## Author’s contribution

Tyng-Shiuan Hsieh: First author, writing.

Yi-Jie Kuo: data collections.

Chen Yu Pin: Corresponding author.

## Registration of research studies

Our manuscript is a case report not a research.

## Guarantor

Yu-Pin Chen.

## Provenance and peer review

Not commissioned, externally peer-reviewed.

## Declaration of Competing Interest

All authors have no conflict of interest.
